# PReS-FINAL-2088: Risk of severe adverse events in juvenile idiopathic arthritis and pediatric-onset inflammatory bowel disease, treated with anti-tnf drugs

**DOI:** 10.1186/1546-0096-11-S2-P100

**Published:** 2013-12-05

**Authors:** P Vatta, A Taddio, L Lepore, V Moressa, S Naviglio, E Prinzi

**Affiliations:** 1 University of Trieste, Trieste, Italy; 2 Institute for Maternal and Child Health-IRCCS "Burlo Garofolo", University of Trieste, Trieste, Italy; 3 Institute for Maternal and Child Health-IRCCS "Burlo Garofolo", Trieste, Italy; 4 "Giuseppe D'Alessandro" Department, University of Palermo, Palermo, Italy

## Introduction

Severe adverse events have been described in children affected by Juvenile Idiopathic Arthritis (JIA) and Inflammatory Bowel Disease (IBD) treated with anti-tnf drugs.

## Objectives

To define the risk of severe adverse events in patients with JIA and IBD treated with anti-tnf drugs.

## Methods

This is a retrospective cohort study. All patients with JIA and IBD attending the "IRCCS Burlo Garofolo" of Trieste from 2000 to 2012 were enrolled. They were divided into 2 groups on the basis of the presence or absence of anti-tnf exposure.

Severe adverse events were considered the followings: a) infections needing anti-tnf permanent suspension and/or hospitalization; b) autoimmune diseases with present or potential organ damage, except for hepatitis and cholangitis during IBD; c) anaphylaxis; d) malignancies.

Univariate analysis testing the effect of anti-tnf exposure on adverse events appearance was realized.

## Results

323 patients were enrolled (159 with JIA and 164 with IBD). 120 patients were exposed to anti-tnf and 203 were not. Infliximab was the most used anti-tnf (73 patients), followed by etanercept (56 patients) and adalimumab (21 patients). Mean total duration of anti-tnf therapy was 26 months (min.1, max.127). The two cohorts were comparable for sex, age, diagnosis and other therapies assumed.

Severe adverse events occurred in 38 anti-tnf-exposed patients (31.7%) and 22 of the not-exposed group (10.8%), with a statistically significant difference (p = 0.000) and a relative risk (RR) of 2.9 (95% confidence interval, CI, 1.8 to 4.7). Anaphylaxis occured in 11 patients (9.2% of the anti-tnf-treated), all assuming infliximab; in the not-treated group none presented reactions (p = 0.000). Infection rate was 6.7% in the anti-tnf-treated group (8 patients) and 3.5% in the not-exposed group (7 patients) (p = 0.273, RR = 1.9, 95% CI: 0.7 to 5.2). Incidence rate of autoimmune diseases in patients treated with anti-tnf was 18.3% (22 patients) vs 7.9% in not-exposed cohort (p = 0.007, RR = 2.3, 95% CI: 1.3 to 4.2). Uveitis was the most frequent autoimmune disease. Both uveitis and lupus-like syndrome were more likely in the subgroup of patients treated with anti-tnf (p = 0.005, RR = 2.5, 95% CI: 1.1 to 6.0 for uveitis and p = 0.050 for lupus-like syndrome). No patients developed malignancies. The outcome of severe anti-tnf drug reactions was as follows: 2 out of 3 uveitis, all anaphylactic reactions, severe infections and lupus-like syndromes healed without organ damage, whereas the other autoimmune complications have been still treating with a good clinical outcome.

## Conclusion

The patients with JIA or IBD treated with anti-tnf have a higher risk of severe adverse events, like anaphylaxis and autoimmune diseases (in particular uveitis and lupus-like syndrome), whereas it seems that this risk does not exist for severe infections. No malignancies were observed during follow up.

Our data suggest that, although the risk of severe adverse reactions to anti-tnf therapy is significant, the occurrence of a permanent damage results very low.

## Disclosure of interest

None declared.

